# A Case of Poisoning by Opium Treated Successfully with Cold Water

**Published:** 1828-07

**Authors:** John F. Brown

**Affiliations:** Jackson, Tennessee


ART. II. *A case of* POISONING *by* OPIUM, *treated with success by the affusion of Cold Water. By* DR. JOHN F. BROWN, *of Jackson, Tennessee.*

On the 30th June, 1825, early in the morning, I was called to see Mr. Harris. On my arrival, I found him lying on his back, apparently in a profound sleep; and was informed, that every exertion had been made to awaken him, but ineffectually. There was no suspicion of his having attempted to destroy himself; and no knowledge of his having taken opium. After examining him for some time, I was confident he was under the influence of some powerful narcotic. His pulse was about 45 in a minute; skin cool and flaccid, slightly moist with clammy sweat; breathing slow and laborious; pupils of the eyes dilated; and his jaws strongly set. I had them opened, and on inspecting the mouth, found some small pieces of opium sticking to his jaw teeth. This confirmed our first impression; and, accordingly, with as much haste as possible, I endeavoured to excite vomiting, and arouse him from his profound lethargy. But all attempts were ineffectual. Fifteen grains of tartar emetic were forced into his stomach, and repeated in 10 or 15 minutes, the fauces being titillated with a feather; but without the least possible effect. The sulphate of zinc was also administered freely, but to no better purpose.

His situation still growing more alarming, I requested the attendance of Doctors Young and Snider. On their arrival, the case was believed to be hopeless, as every exertion to arouse the system had proved ineffectual. It was immediately determined to use the cold affusion, in imitation of Doctors Jackson and Cross. We, accordingly, had him placed on the floor, and poured a large vessel of cold water on him, commencing with his face and directing it to his breast. The first vessel was expended without producing any sensible effect. We resorted, without delay, to a second, which appeared to produce some effect, for he began to struggle. So soon as we began pouring the third, he suddenly raised himself on his nates and said, "I cannot stand every thing." We continued the cold affusion, at the same time endeavouring to excite vomiting by titillating the fauces, which was effected so soon as sensation was awakened. As soon as practicable, we directed strong coffee to be taken freely. In about two hours the emetic medicines produced a free catharsis. We directed frictions and moderate exercise, with some other gentle remedies, intended to obviate the effects of the opium, and left him.

I did not visit my patient until late in the evening, when, to my astonishment, I found him cheerful, and apparently free from the effects of the opium. All the secretions appeared to be completely reestablished. I directed him to keep his bowels open with salts or oil. He continued to mend, and was free from all symptoms of the poisoning in a few days.

From the statement of the patient, after his recovery, it must have been 14 hours from the time he swallowed the opium, until I saw him. He said he had swallowed a large piece at bed time, the night before. The first dose made him vomit; and, thinking he had thrown up the principal part, he repeated the portion. From the description of this piece, it must have weighed two drams, both together making half an ounce. Whether he threw up the whole of the first dose, is not known.

This case would seem to be of considerable importance, when we take into consideration the period which elapsed from the time of taking the opium, until medical aid could be had,—and his very quick recovery, without the train of disagreeable symptoms, which are not unusual, when large quantities of opium have been taken.

*Jackson, Tennessee, May* 27, 1828.

